# *cmdABCDEF*, a cluster of genes encoding membrane proteins for differentiation and antibiotic production in *Streptomyces coelicolor *A3(2)

**DOI:** 10.1186/1471-2180-9-157

**Published:** 2009-08-04

**Authors:** Pengfei Xie, Ana Zeng, Zhongjun Qin

**Affiliations:** 1 Key Laboratory of Synthetic Biology, Shanghai Institute of Plant Physiology and Ecology, Shanghai, PR China

## Abstract

**Background:**

*Streptomyces coelicolor *is the most studied *Streptomyces *species and an excellent model for studying differentiation and antibiotic production. To date, many genes have been identified to be required for its differentiation (e.g. *bld *genes for aerial growth and *whi *genes for sporulation) and antibiotics production (including *actII-orf4*, *redD*, *cdaR *as pathway-specific regulatory genes and *afsR*, *absA1/A2 *as pleiotropic regulatory genes).

**Results:**

A gene cluster containing six genes (*SCO4126-4131*) was proved to be co-transcribed in *S. coelicolor*. Deletions of *cmdABCDEF *(*SCO4126-4131*) displayed defective sporulation including formation of aberrant branches, and abnormalities in chromosome segregation and spore septation. Disruption mutants of apparently orthologous genes of *S. lividans *and *S. avermitilis *also showed defective sporulation, implying that the role of these genes is similar among *Streptomyces*. Transcription of *cmdB*, and therefore presumably of the whole operon, was regulated developmentally. Five of the encoded proteins (CmdA, C, D, E, F) were predicted membrane proteins. The other, CmdB, a predicted ATP/GTP-binding protein with an ABC-transporter-ATPase domain shown here to be essential for its function, was also located on the cell membrane. These results indicate that CmdABCDEF proteins mainly affect *Streptomyces *differentiation at an early stage of aerial hyphae formation, and suggest that these proteins may form a complex on cell membrane for proper segregation of chromosomes. In addition, deletions of *cmdABCDEF *also revealed over-production of blue-pigmented actinorhodin (Act) via activation of transcription of the pathway-specific regulatory gene *actII-orf4 *of actinorhodin biosynthesis.

**Conclusion:**

In this study, six co-transcribed genes *cmdABCDEF *were identified by their effects on differentiation and antibiotic production in *Streptomyces coelicolor *A3(2). These six membrane-located proteins are possibly assembled into a complex to function.

## Background

*Streptomyces *are Gram-positive eubacteria that are the major natural source of antibiotics, producing about half of all known microbial antibiotics [1]. This genus also has a complex life cycle, in which spores germinate to form a substrate mycelium of branching hyphae on solid medium, from which branches grow into the air, such multi-nucleoid aerial hyphae ultimately becoming septated to form chains of unigenomic spores [2,3].

*Streptomyces coelicolor *is the most studied *Streptomyces *species and an excellent model for studying antibiotic production and differentiation [4]. It produces several chemically different antibiotics, including the blue-pigmented actinorhodin (Act), red-pigmented undecylprodigiosin (Red), calcium-dependent antibiotic (CDA) and plasmid SCP1-encoded methylenomycin (Mmy). Pathway-specific regulatory genes, e.g. *actII*-*orf4*, *redD*, *cdaR *and *mmyB*, are required for initiating transcription of the corresponding antibiotics biosynthetic gene clusters; while pleiotropic regulators, e.g. AfsR, often affect multiple secondary metabolism [5,6]. By using *S. coelicolor *as a model system, two dozen genes (*bld *and *whi*), most of them encoding regulatory proteins, important for initiation of aerial mycelium formation and sporulation have been identified [7]. More than 20 other genes from primary metabolism (e.g. *citA *encoding citrate synthase; [8]) and stress-response (*rsrA *for oxidation-sensing anti-sigma protein; [9]) etc also affect *Streptomyces *differentiation, indicating that the regulatory signaling cascades for aerial growth and sporulation extensively interact with metabolic, morphological, homeostatic and stress-related checkpoints [10]. Recently, several key genes affecting apical growth, chromosome segregation and cell division (e.g. *divIVA, sffA, ftsZ, ftsQ, ftsK *and *parA/B*; [11-17]) have been identified.

Here we describe identification of a cluster of six co-transcribed genes *cmdABCDEF *(encoding five membrane proteins and one membrane-located ATP/GTP-binding protein) in *S. coelicolor *that affect sporulation and antibiotic production.

## Results

### Co-transcription of six genes *SCO4126-4131 *of *S. coelicolor*

**Figure 1:**
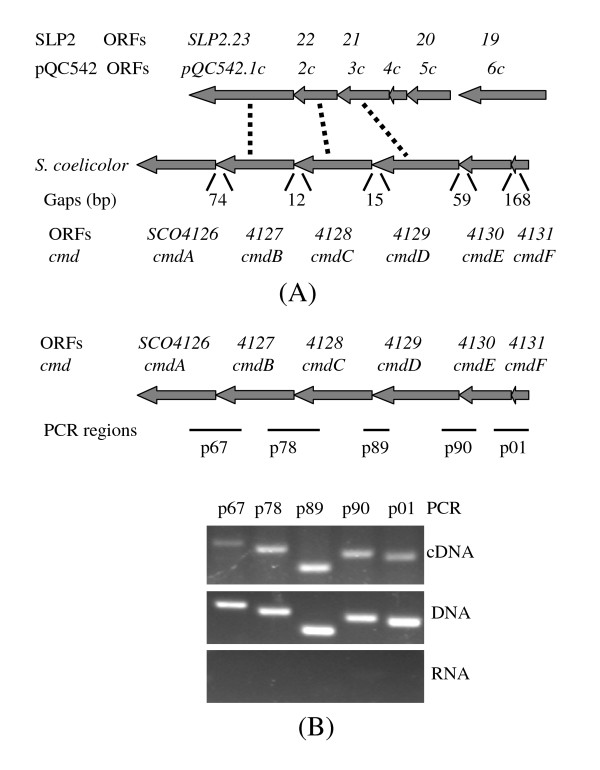
**Organization and transcription of the six genes *SCO4126-4131 *of *S. coelicolor***. (A) Comparison of organization of the *SCO4126-4131 *genes of the *S. coelicolor *chromosome and the *SLP2.19-23 *(or *pQC542.1c-6c*) genes of *S. lividans *plasmid SLP2. The homologous genes are indicated by dashed lines and transcriptional directions of genes by filled arrowheads. (B) RT-PCR of transcript overlapping the consecutive adjacent genes of the *SCO4126-4131 *cluster. RNA of strain M145 was isolated and reverse-transcribed into cDNA. The cDNA, RNA and M145 chromosomal DNA were used as templates. Five paired primers (i.e. p67, p78, p89, p90 and p01) were used to allow amplification of segments extending from each gene into its immediate neighbor. PCR products were electrophoresed in 2% agarose gel at 100 v for 1 h.

Earlier work indicated that the six co-transcribed genes (*SLP2.19-23 *or *pQC542.1c-6c*) of *Streptomyces *linear plasmid SLP2 are required for plasmid conjugal transfer [18,19]. Interestingly, three genes *SLP2.21-23 *resembled *SCO4127-4129 *of *S. coelicolor *chromosome (identities were 33% [133/393], 29% [56/193] and 22% [97/435] respectively), which were also located in a cluster of six genes *SCO4126-4131 *(Figure 1A). The transcription directions of *SCO4126-4131 *were same. To see if they were co-transcribed, reverse transcription-PCR (RT-PCR) was employed. As shown in Figure 1B, compared with the positive (genomic DNA as template for PCR reaction) and negative controls (total RNA as template), the expected sizes of PCR products were detected on agarose gel from the cDNA, reversely transcribed from the total RNA, by using primers from the neighboring genes of *SCO4126-4131*. While this analysis does indicate a transcript exists that covers the entire length of the cluster, it is possible that other transcripts exist from other promoters within the cluster that do not span all 6 genes.

To investigate if *SCO4126-4131 *were involved in plasmid transfer, null mutants of the whole gene cluster were constructed by PCR-targeted mutagenesis [20]. However, no significant difference in transfer frequencies of the SLP2-derived linear plasmid pQC542 which contained genes for DNA replication in linear mode and plasmid conjugal transfer [18,19] between the mutant and the wild-type was found (data not shown), suggesting that these chromosomal genes could not substitute for the SLP2 genes for plasmid transfer.

### Null mutants of *SCO4126-4131 *display defective sporulation

**Figure 2:**
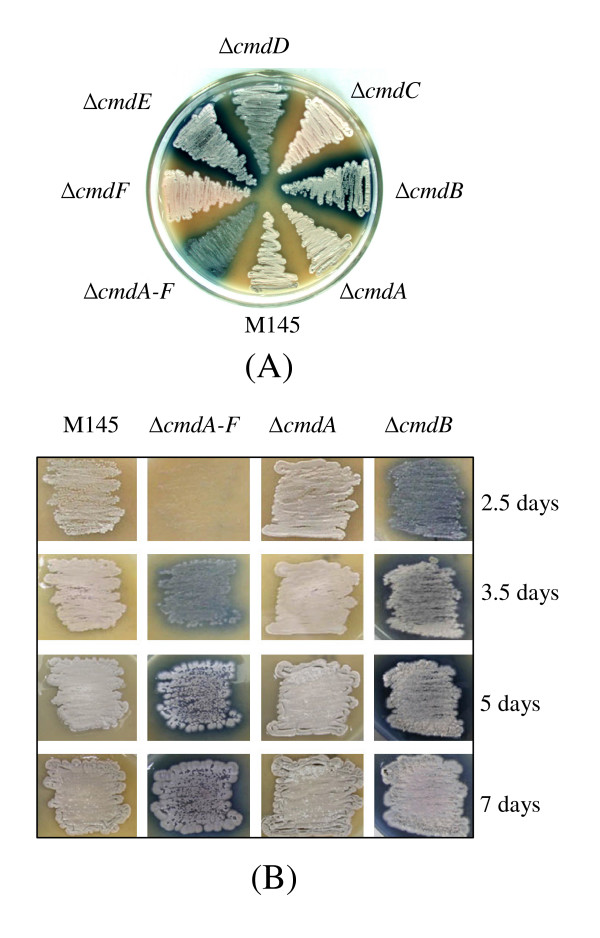
**Phenotype of the null mutants of *cmdABCDEF *on MS plates**. (A) Growth of single and multiple null mutants of the *cmdABCDEF *genes on MS for three days. The parental strain is M145. (B) A time course of culturing M145 and the null mutants. Strains were inoculated as ~1 cm^2 ^patches on MS medium. Time points of observation are shown on the right.

To study the functions of *SCO4126-4131*, null mutants of the individual genes or complete gene cluster were constructed by in-frame replacement via PCR-targeting with an apramycin resistance gene and then removing the marker, excluding potential polar effects on expression of the gene cluster. After culturing the mutants on MS medium for 3 days, as seen in Figure 2A, the Δ*SCO4126 *strain, as well as wild-type strain M145, produced dark grey colonies on agar plate, whereas colonies of all the other null mutants, including a Δ*SCO4126-4131 *mutant, were light grey, and seemed to produce fewer spores. In time courses of M145 and null mutants of *SCO4126, SCO4127 *and *SCO4126-4131 *on MS agar (Figure 2B), the Δ*SCO4127 *or Δ*SCO4126-4131 *strains had a significant delay in aerial mycelium formation, and sporulated 1 or 2 days later than the wild-type strain, while there was no apparent difference in sporulation between M145 and the Δ*SCO4126 *strain. Introduction into the mutants of the corresponding genes or the whole gene cluster by derivatives of the chromosomally integrating plasmid pSET152 or pFX101 could restore the timing of forming aerial mycelium and sporulations to the wild-type level, but introduction of the *SCO4127 *alone could not complement the defects in sporulation in the *SCO4126-4131 *mutant (data not shown), confirming that the observed defective sporulation was caused by deletions of the genes. Five of the *SCO4126-4131 *genes encoded membrane proteins, while *SCO4127 *encoded an ATP/GTP-binding protein. Thus, the *SCO4126-4131 *gene cluster was designated *cmdA-F *(a cluster of genes encoding membrane proteins for differentiation).

### Aberrant branches, defective spore septation and abnormal chromosome segregation in null mutants

**Figure 3:**
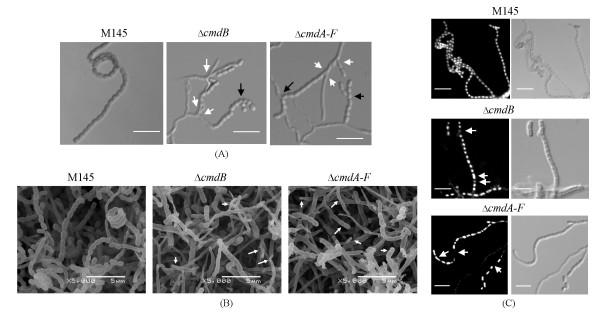
**Observation of sporulating aerial hyphae by phase contrast, fluorescence and electron microscopy**. (A) Abnormal branches at the aerial hyphae of the mutant observed by contrast microscopy. The Δ*cmdB *and Δ*cmdA-F *mutants frequently produced multiple branches in aerial hyphae, both low in the hyphae (indicated by white arrows), and near the tips (black arrows). These are not common in the wide-type M145. Size bars correspond to 5 μm. (B) Observation of spores in M145 and null mutants of *cmdB *or *cmdA-F *under scanning electron microscopy. Strains were inoculated on MS medium covered with cellophane at 30°C for 7 days. Samples were treated (Materials and methods) and subjected to SEM observation. The collapsed aerial hyphae and short spore chains are indicated by white arrows. (C) Chromosomes in the aerial hyphae were stained by DAPI, and observed by laser-scanning confocal microscopy. The chromosomes were not normally segregated in some of the pre-spores of the mutants, some compartments receiving none and some containing more than one chromosome (indicated by white arrows).

After harvesting, diluting and plating out spores on medium, the numbers of spores (c. 10^6^/ml) obtained from the Δ*cmdB *and especially Δ*cmdA-F *strains were obviously less than that of wide type M145 (c. 10^8^/ml). To characterise these aerial hyphae and spores, we employed phase-contrast and scanning electron microscopy. Under phase-contrast microscopy, normally long unbranched aerial hyphae were seen in M145, whereas multiple branching from both aerial and apical hyphae, giving rise to unusually short spore chains, was observed in the Δ*cmdB *and Δ*cmdA-F *strains (Figure 3A). Scanning electron microscopy revealed, in contrast to nearly complete septation of aerial hyphae and formation of abundant long spore chains in M145, most aerial hyphae in null mutants of *cmdB *and *cmdA-F *were collapsed and unable to septate to become spores, while some of hyphae could eventually develop into short spore chains (Figure 3B). To further dissect these sporulating aerial hyphae, we employed fluorescence microscopy. Sporulating hyphae were fixed and then their chromosomes were stained with 4',6-diamidino-2-phenylindole (DAPI). Fluorescence microscopy revealed that chromosomes in wide-type M145 were distributed at regularly spaced intervals along spore chains (Figure 3C), and anucleate spores were observed at a low frequency (0.1%, c.1000 spores counted). However, incomplete separation of chromosomes was readily seen in the mutants, shown as unevenly stained chromosomes along spore chains (Figure 3C); and anucleate spores appeared at a frequency of 8% and 6% along spore chains for the Δ*cmdB *and Δ*cmdA-F *strains (c.500 spores counted), respectively. Taken together, the Δ*cmdB *or Δ*cmdA-F *strains showed aberrant branches, defective chromosome segregation and abnormally spaced spore septation.

### CmdB, an ATP/GTP-binding protein with an ABC-transporter ATPase domain, is located on the cell membrane

**Figure 4:**
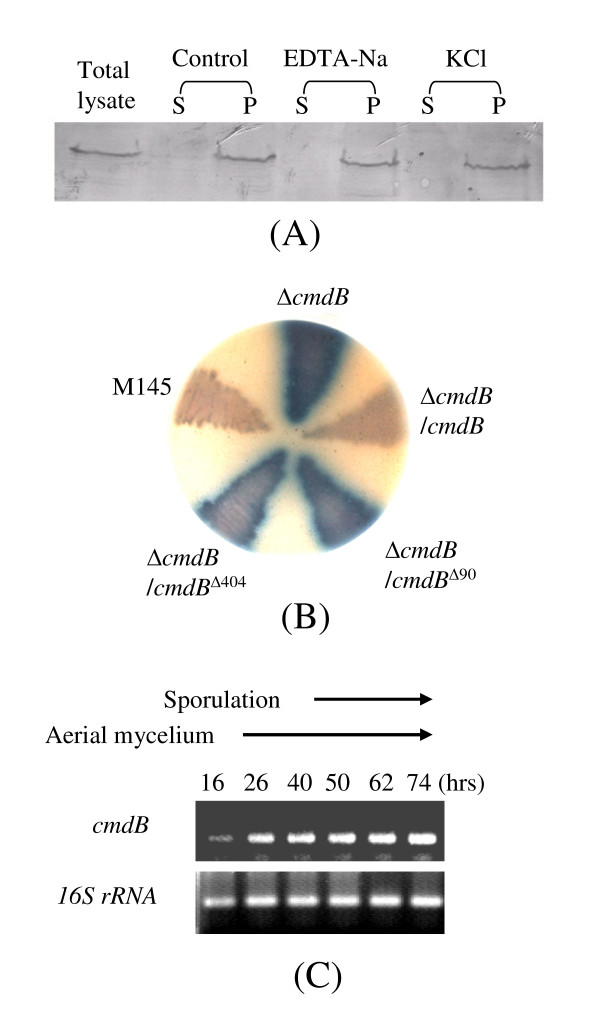
**Localization of CmdB protein, characterization of its functional domain, and detection of *cmdB *transcription**. (A) Localization of CmdB protein. Cell lysates of strain M145 and that were treated with 0.5 M KCl or 5 mM EDTA-Na, were centrifuged to obtain supernatants (S) and pellets (P) for Western blotting with CmdB polyclonal antibody. Total cell lysates was a positive control. (B) Mutations of conserved residues in domains of the CmdB protein blocked its function. Plasmid pFX101 derivatives containing the site-mutated *cmdB *genes were introduced by conjugation into the *cmdB *null mutant. Strains were grown on MS at 30°C for 3 days. (C) RT-PCR to detect transcription of *cmdB*. Total RNA was isolated from MS medium grown for 16, 26, 40, 50, 62 and 74 h, and reverse-transcribed into cDNAs for PCR amplification. Transcription of *16S rRNA *gene was used as an internal control.

*cmdB *encoded an ATP/GTP-binding protein and *cmdA, C, D, E and F *encoded membrane proteins. To see if CmdB protein was also located on the cell membrane, both membrane and cytoplasmic fractions were prepared from cell extracts, electrophoresed on a denatured polyacrymide gel and probed by Western-blotting with anti-CmdB antibody. As seen in Figure 4A, CmdB protein was only detected in membrane (precipitate) but not in cytosolic (supernatant) fractions.

CmdB contained an ABC-transporter-ATPase domain (from positions 44 to 427) according to Superfamily 1.69 analysis http://supfam.mrc-lmb.cam.ac.uk/SUPERFAMILY/hmm.html. This superfamily includes several families of characterized or predicted ATPases which are predominantly involved in extrusion of DNA and peptides through membrane pores [21]. To investigate whether this domain was required for the function of CmdB, lysines at conserved positions 90 or 404 were mutated to arginines by site-directed mutagenesis (K90A or K404A). The mutated *cmdB *genes were cloned into pFX101, and then introduced by conjugation into the *cmdB *null mutant. In contrast to the functional *cmdB *gene, the site-mutated *cmdB *genes could not complement the *cmdB *null mutant to reverse its phenotype of over-production of blue pigment (Figure 4B) and also to produce dark grey colony to the wild type level (data not shown). These results indicated that the mutated residues were essential for function. It was however also possible that the mutations had destabilised the protein, causing it to degrade much more rapidly than the wild-type form.

### Transcription of *cmdB *during differentiation

To see if transcription of *cmdB *was regulated during differentiation, strain M145 grown on MS medium was harvested at different times for RT-PCR and analysed using primers specific for *cmdB*. As seen in Figure 4C, a small amount of *cmdB *transcript could be detected from mainly vegetative mycelium (16 h), and a larger amount (at least five-fold) was produced at the stage of aerial mycelium formation (26 h) and continued to increase during sporulation (40–74 h). These results suggested that transcription of *cmdB *was regulated temporally, possibly developmentally.

### The *cmdA-F *orthologues in *S. lividans and S. avermilitis *also affect differentiation

**Figure 5:**
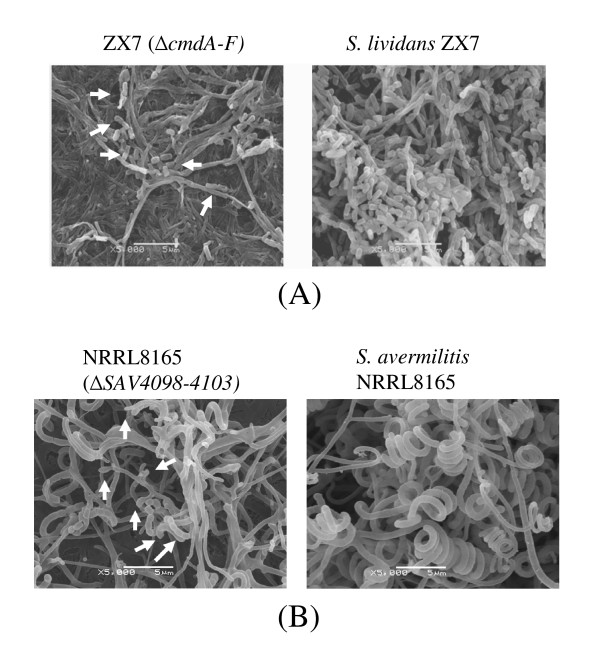
**Observation of the null mutants of *cmdA-F *orthologues in *S. lividans *and *SAV4098*-*4103 *genes in *S. avermitilis *under scanning electron microscopy**. (A) *S. lividans *ZX7 and its *cmdA-F *null mutant were cultured on MS at 30°C for 5 days, and then subjected to observation by scanning electron microscopy. The mutant produced less abundant aerial mycelium, most of which consisted of relatively short spore chains (white arrows). (B) Observation of *S. avermitilis *NRRL8165 and a null mutant of the *SAV4098*-*4103 *genes. Short aerial hyphae are indicated by white arrows.

By using primers from *cmdA-F *of *S. coelicolor *M145 and template DNA from *S. lividans *ZX7, the same sizes of PCR bands as M145 were detected (data not shown), suggesting that the *S. lividans *genome contained similar genes. The cosmid used in constructing the *cmdA-F *null mutant of M145 was introduced by conjugation into ZX7, and the resulting strain displayed a phenotype of very poor sporulation but no visible blue pigment on MS agar plate after culturing for 5 days. A serious block of formation of aerial hyphae in the null mutant was observed under scanning electron microscopy (Figure 5A).

The complete nucleotide sequence of *S. avermitilis *genome reveals a highly homologous gene cluster (i.e. *SAV4098 *to *SAV4103*) to *cmdA-F *[22]. A null mutant of *SAV4098*-*4103 *was constructed in *S. avermilitis *NRRL8165. Its defective sporulation was displayed on MS medium, and blocking in development of coiled aerial hyphae was observed under microscopy compared with that of the wild type (Figure 5B). No over-production of antibiotic avermectin was detected in the null mutant (data not shown).

### Several null mutants of *cmdABCDEF *reveal over-production of blue pigment

**Figure 6:**
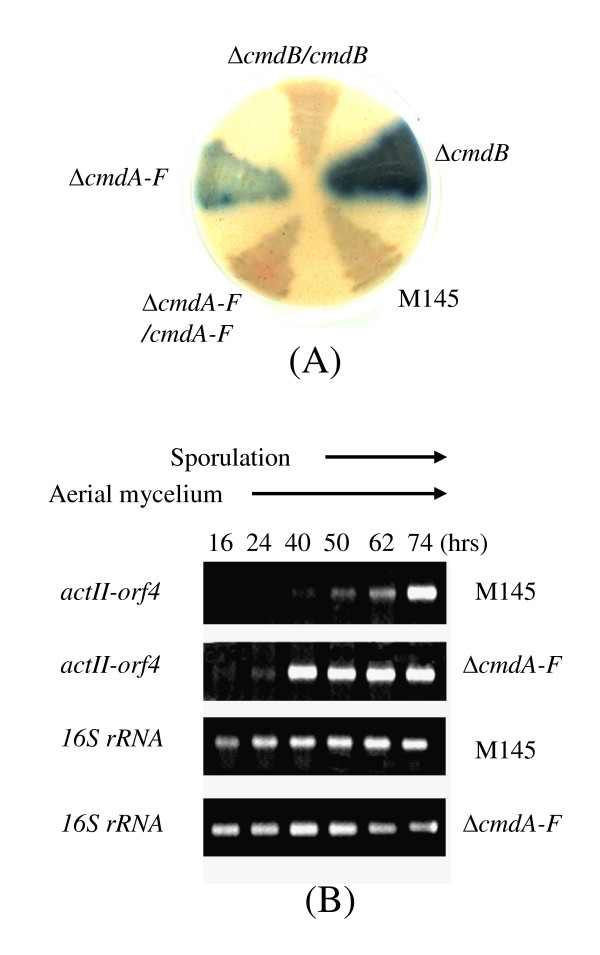
**Observation of blue-pigment overproduction by the null mutants and transcriptional assay of *actII-orf4 *of the actinorhodin biosynthetic gene cluster**. (A) Blue-pigment over-production by the null mutants of *cmdB *or *cmdA-F *and complementation by introduction of the corresponding functional genes. Strains were grown on MS for 3 days at 30°C. The back of the plate is shown. (B) Transcription of *actII-orf4 *in null mutant of *cmdA-F*. Total RNA was isolated from solid MS cultures grown for 14, 24, 50, 62, and 74 h, and reverse-transcribed into cDNA for PCR amplification. The *16S rRNA *gene of the mutant was used as an internal control. PCR products were electrophoresed in 2% agarose gel at 100 v for 0.5 h.

As seen in Figure 2A, null mutants of *cmdB, D, E *or *cmdA-F *also produced a large amount of blue pigment on MS medium, while little or no blue pigment was produced for other null mutants (i.e. *cmdA, C *and *F*) and wild type M145. Introduction of additional copies of the functional *cmdB *or *cmdA-F *into the mutants could reduce the production of blue pigment to the wild-type level (Figure 6A), confirming that blue pigment over-production was caused by mutation of the genes, and also suggesting that these genes are involved in repression of blue pigment production in M145.

### Initiating transcription of the pathway-specific regulatory gene *actII-orf4 *of actinorhodin biosynthesis at an earlier growth stage in the *cmdA-F *null mutant

In *S. coelicolo*r, pathway-specific regulatory gene *actII-orf4 *is essential for initiating transcription of the whole biosynthetic gene cluster of blue-pigment actinorhodin [23]. To study transcription of *actII-orf4 *in the *cmdA-F *null mutant, we harvested spores/mycelium from MS plates after different growth periods and isolated RNA for RT-PCR. As seen in Figure 6B, transcription of *actII-orf4 *in the null mutant started as early as 16 h and then reached a maximum at 40 h, ~24 and 34 h earlier than was observed in M145.

## Discussion

Here, we report that an operon of six genes *cmdABCDEF *(*SCO4126-4131*) of *S. coelicolor*, encoding five membrane proteins and one membrane-located ATP/GTP-binding protein, affects differentiation and causes increased production of an antibiotic, actinorhodin. The Δ*cmdABCDEF *strains reveal aberrant branches and short aerial hyphae. Expression of *cmdB*, and therefore presumably of the whole operon, was detectable during vegetative growth, but increased substantially as soon as aerial growth was detectable. Similar conserved gene clusters are also found in other *Streptomyces *species, e.g. *S. avermitilis *(*SAV4098*-*4103; *[22]), *S. griseus *(*SGR3915-3920*; [24]) and *S. lividans *(Our unpublished data). Serious block in forming aerial hyphae in *S. lividans *and in the development of coiled aerial hyphae in *S. avermitilis *were observed when their *cmd *operons were disrupted. Together, these results indicate that CmdABCDEF proteins mainly affect *Streptomyces *differentiation early in aerial hyphae formation.

The Δ*cmdABCDEF *strains of *S. coelicolor *also showed defective chromosome segregation during sporulation. In prokaryotes, motor proteins such as FtsK and SpoIIIE containing a conserved RecA domain are often associated with DNA translocation during processes of cell division, conjugation and sporulation [25]. In *S. coelicolor*, FtsK and ParA/ParB are required for proper chromosome segregation during sporulation [15,16]. However, despite detectable levels of errors in chromosome segregation in FtsK or ParAB mutants, the majority of chromosomes still appear to segregate properly, suggesting that other proteins are also involved in chromosome partition or segregation. According to analysis using the Protein Homology/analogY Recognition Engine PHYRE http://www.sbg.bio.ic.ac.uk/phyre/html/index.html, CmdB protein was predicted containing a RecA domain (from positions 77 to 407, expectation value 1.7 × 10^-21^) or *E. coli*-FtsK motor domain (3.3 × 10^-12^), suggesting that it might be an ATP/GTP-dependent motor protein. CmdB displays homology with VirB4-like proteins from *Frankia*, *Brevibacterium*, *Geobacillus *and *Thermoanaerobacter *(expectation values 3 × 10^-42^, 1 × 10^-39^, 7 × 10^-9 ^and 2 × 10^-9^, respectively) etc. The VirB4, an essential component of the bacterial type IV system, interacts with other membrane proteins in the *vir *operon to assemble a pore for transfer of a DNA-protein complex [26,27]. Since CmdB is also located on the cell membrane, it is likely that CmdB along with other five membrane proteins from the same gene cluster might form a complex on the cell membrane. Further study will be needed to explore the existence of such a complex and to investigate whether it could form a type IV-like channel on cell membrane for chromosome and/or plasmid translocation in *Streptomyces*.

About 836 and 69 genes of *S. coelicolor *genome are predicted to encode membrane and ATP/GTP-binding proteins, respectively ([28]; http://www.sanger.ac.uk/Projects/S_coelicolor/classwise.html#class4.1.0). Among these, *SCO6878*, *SCO6880 *and *SCO6881*, located in a cluster of 14 probably co-transcribed genes *SCO6871-6884*, highly resemble *cmdB*, *cmdC *and *cmdD*, respectively. However, null mutants of *SCO6878 *or *SCO6881 *did not display defective sporulation or over-production of blue pigment on MS medium (our unpublished data). Thus, either these genes are not involved in sporulation and antibiotic production, or their role may be masked by functional overlap with other genes, or the phenotype might be manifested only under particular conditions.

## Conclusion

This study describes the identification of six co-transcribed genes *cmdABCDEF*, deletions of which displayed over-expression of blue-pigmented Act, defective sporulation and especially abnormalities in chromosome segregation, indicating that *cmdABCDEF *are new genes involved in antibiotic production and differentiation of *S. coelicolor*.

## Methods

### Bacterial strains, plasmids and general Methods

*S. coelicolor *M145 [28], *S. lividans *ZX7 [29] and *S. avermitilis *NRRL8165 [22] were hosts for studying functions of *cmdABCDEF *genes. *Streptomyces *were cultivated on Mannitol Soya flour medium (MS; 30). A cellophane sheet was placed over the agar medium when it was necessary to collect mycelium/spores or when cultures were to be examined by scanning electron microscopy [31]. Manipulation of *Streptomyces *DNA and RNA followed Kieser *et al*. [30]. *E. coli *strain DH5α (Life Technologies Inc) was used as cloning host. Plasmid isolation, transformation and PCR amplification followed Sambrook *et al*. [32]. DNA fragments were purified from agarose gels with the Gel Extraction Master kit (Watson).

### Construction and complementation of *Streptomyces *null mutants

Cosmid SCD72A of *S. coelicolor *containing *cmdABCDEF *genes was kindly provided by Professor David Hopwood. Cosmid SAV3-17 of *S. avermitilis *containing the *SAV4098*-*4103 *genes was constructed in our laboratory. PCR-targeted mutagenesis was used to replace precisely the *cmdABCDEF *or *SAV4098*-*4103 *genes with an antibiotic resistant gene and then remove the marker but leaving an 81-bp "scar" sequence when necessary [20]. Derivatives of the *Streptomyces *chromosomal-integrating plasmid pSET152 [33] or pFX101 containing the functional *cmdABCDEF *genes were employed for complementing the mutated genes. PCR primers for construction and complementation of *Streptomyces *null mutants are listed in Additional file 1.

### Scanning electron microscopy (SEM)

*Streptomyces *cultures were grown on MS medium covered with cellophane disks. After 7 days incubation at 30°C, the cells were fixed with fresh 2% glutaraldehyde (pH7.2) and 1% osmium tetroxide. After dehydration, ethanol was replaced by amyl acetate. The samples were then dried with the supercritical drying method in HCP-2 (Hitachi), coated with gold by Fine Coater JFC-1600 (Jeol), and examined with a JSM-6360LV scanning electron microscopy (Jeol).

### Light microscopy

*Streptomyces *spores were evenly spread onto MS medium, into which cover-slips were then inserted at an angle of approximate 60°C. After 4 days incubation at 30°C, cells attached to cover-slips were fixed with methanol followed by washing with phosphate-buffered saline. Samples were then stained with 4',6-diamidino-2-phenylindole (DAPI, 25 μg/ml) at room temperature for 30 minutes. After that, samples were observed by laser scanning confocal microscope Fluoview FV1000 (Olympus). Images were processed with Image-Pro Plus 6.0.

### Reverse-transcription (RT) PCR assay

*S. coelicolor *were cultured on MS medium covered with cellophane disks, and RNA was isolated from cultures at a series of incubation times. The RNA samples were treated with DNase (RNase-free, Takara) to remove possible contaminating DNA and, after quantification, reverse-transcribed into cDNA by using "Revert Acid First Strand cDNA Synthesis" kit (MBI Fermentas). Then equal 25-ng products were subjected to PCR amplification (25 cycles). Five paired primers (p67, p78, p89, p90 and p01; see Additional file 2) were used for validating co-transcription of the *cmdABCDEF *genes. Three paired primers, Pact, PcmdB and P16S (Additional file 2), were used to detect transcription levels of *actII-orf4, cmdB *and genes for 16S rRNA, respectively. PCR conditions were: template DNA denatured at 94°C for 5 min, then 94°C 30 s, 60°C 30 s, 72°C 50 s, for 25 cycles.

### Site-directed mutagenesis of *cmdB*

The site-directed mutagenesis of *cmdB *was performed by using the QuikChange kit (Stratagene). Plasmid pFX103 containing the intact *cmdB *and promoter of *cmdABCDEF *was used as PCR template. Two paired primers, PcmdBK90A (5'-tcggtgatcaggtgtctgaccacctggacgt-3', 5'-acgtccaggtggtcagacacctgatcaccga-3') and PcmdBK404A (5'-Tctcgagggccgacctgccgttccccgactc-3', 5'-Gagtcggggaacggcgagtcggccctcgaga-3'), were used to change lysines of CmdB at positions 90 and 404 into arginines.

### CmdB protein and Western blotting

The PCR-amplified *cmdB *gene was cloned between the *Eco*RI and *Bam*HI sites of *E. coli *plasmid pET-28a (Novagen), and the resulting plasmid was introduced by transformation into *E. coli *strain BL21 (DE3). Over-expression of CmdB was induced by adding 1 mM isopropyl-β-D-thiogalactopyranoside (IPTG) at 20°C for 12 hours. The six-histidine-tagged CmdB was purified by Ni^2+ ^column chromatography (Qiagen) and used to raise rabbit polyclonal antibodies (the Antibody Center of the Shanghai Institutes for Biological Sciences).

*S. coelicolor *M145 was cultivated in Typtone-Soya-Broth medium [30] for 24 hours. Cells were sonicated and debris was removed by centrifugation (12,000 × g, 10 min). Then the lysate was incubated with 0.5 M KCl or 5 mM EDTA at 4°C for 30 min, prior to separation into cytosolic (supernatant) and membrane (precipitate) fractions by ultracentrifugation at 180,000 × g for 2 h [34]. Each fraction together with the cell lysate was electrophoresed in a 12% SDS-polyacrylamide gel, and then transferred onto a PVDF membrane (Immobilon-P, Millipore) by electrophoresis. The PVDF film was incubated with the polyclonal antibody and horse-radish peroxidase-conjugated anti-rabbit IgG (Amersham). After 3 times washing, the signal on the film was directly detected by HRP Substrate Reagent (Shenergy).

## Authors' contributions

PFX conceived of the entire study, performed most of the experiments including gene (s) disruption, protein expression/purification, western blotting, microscopy, RT-PCR, and also drafted the manuscript. AZ performed disruption of genes in *S. lividans *ZX7. ZJQ was involved in project design, and prepared the manuscript. All authors discussed the results and assisted with editing of the manuscript.

## Supplementary Material

Additional file 1 **PCR primers for construction and complementation of *Streptomyces *null mutants**. The PCR primers listed were used to construct or complement the *Streptomyces *null mutants. Click here for file

Additional file 2 **Primers for reverse-transcription (RT) PCR**. The PCR primers listed were used to verify the co-transcription of *cmd *operon or detect the gene expression at the transcriptional level. Click here for file
