# Supplementary material for: cmdABCDEF, a cluster of genes encoding membrane proteins for differentiation and antibiotic production in Streptomyces coelicolor A3(2)
Source: BMC Microbiol. 2009 Aug 4;9:157. doi: 10.1186/1471-2180-9-157 (PMC2782261; doi:10.1186/1471-2180-9-157)
Supplement: Additional file 1 — PCR primers for construction and complementation of Streptomyces null mutants. The PCR primers listed were used to construct or complement the Streptomyces null mutants. [file 1471-2180-9-157-S1.pdf]

Table 1S. PCR primers for construction and complementation of *Streptomyces* null mutants

Primer name	Sequences (from 5′ to 3′)	Mutated strains
Pmtsco4126	CCTGGCGCTCCTGCTGCGCCGGTGCTCCTACTGGCCCTATGTAGGCTGGAGCTGCTTC	*S. coelicolor* △*cmdA*
	TTCGCCACCGGCATCCGAAGGTAGGGGCTGAGCCTGATGATTCCGGGGATCCGTCGACC	
Pmtsco4127	CCTGGCGCTCCTGCTGCGCCGGTGCTCCTACTGGCCCTATGTAGGCTGGAGCTGCTTC	*S. coelicolor* △*cmdB*
	TTCGCCACCGGCATCCGAAGGTAGGGGCTGAGCCTGATGATTCCGGGGATCCGTCGACC	
Pmtsco4128	GAGGACGGTCAGCGGGTCCCGCATCAGGCTCAGCCCCTATGTAGGCTGGAGCTGCTTC	*S. coelicolor* △*cmdC*
	CACAGGAGGTGACGGGCGTTGACGACCGAGTCCCACATGATTCCGGGGATCCGTCGACC	
Pmtsco4129	GGGGCGTGACCGTATGGGACATGTGGGACTCGGTCGTCATGTAGGCTGGAGCTGCTTC	*S. coelicolor* △*cmdD*
	ACCGCTTTGGTTGCCGTTGCTCAGGCAGCCGCCGTCGTGATTCCGGGGATCCGTCGACC	
Pmtsco4130	GCAACCAAAGCGGTGAGTTTGAGCGCGCGGTGCTGGCTATGTAGGCTGGAGCTGCTTC	*S. coelicolor* △*cmdE*
	ACACGGCCGACCCGACGTCGAGGGGGCGTACGCGGCATGATTCCGGGGATCCGTCGACC	
Pmtsco4131	GTGGGATGGAGGGGGAGGAACGGTTGCGTGCCGGGGTTATGTAGGCTGGAGCTGCTTC	*S. coelicolor* △*cmdF*
	AGGGCGCGGAACTACCCAACAGGGGCGGTGACTTACATGATTCCGGGGATCCGTCGACC	
Pmtsco4126-31	CCTGGCGCTCCTGCTGCGCCGGTGCTCCTACTGGCCCTATGTAGGCTGGAGCTGCTTC	*S. coelicolor* △*cmdA-F*
	AGGGCGCGGAACTACCCAACAGGGGCGGTGACTTACATGATTCCGGGGATCCGTCGACC	
Pmtsco6878	GGTGTGCACGTCCCGCCCCCGGGAGTTGGTCAGGGGTCATGTAGGCTGGAGCTGCTTC	*S. coelicolor* △*SCO6878*
	CCCGCGCCTGACACAAGAAAGGAGCAGTAACTCCCCGTGATTCCGGGGATCCGTCGACC	
Pmtsco6881	TGTCGCGAGTGGTCTGGAATGAACCCATTTCGGGTTTCATGTAGGCTGGAGCTGCTTC	*S. coelicolor* △*SCO6881*
	CGCGATGAGTACCACCCGCAGAATCGAAGGCCCCCGATGATTCCGGGGATCCGTCGACC	
Pmtsav4098-4103	AGGGCGCAGAACTACCCACTAGGGGCGGTGACTTACATGATTCCGGGGATCCGTCGACC	*S. avermilitis SAV4098-4103*
	GGCTGCTCCGCGCCGCTGCCGGGGCGAAGGGCCTTGTCATGTAGGCTGGAGCTGCTTC	
