# Supplementary material for: cmdABCDEF, a cluster of genes encoding membrane proteins for differentiation and antibiotic production in Streptomyces coelicolor A3(2)
Source: BMC Microbiol. 2009 Aug 4;9:157. doi: 10.1186/1471-2180-9-157 (PMC2782261; doi:10.1186/1471-2180-9-157)
Supplement: Additional file 2 — Primers for reverse-transcription (RT) PCR. The PCR primers listed were used to verify the co-transcription of cmd operon or detect the gene expression at the transcriptional level. [file 1471-2180-9-157-S2.pdf]

Table 2S. Primers for reverse-transcription (RT) PCR

Primer name	Sequences (from 5′ to 3′)	Features
P67	GTGCCCATCACGAACACCGT<br>TGATCACCGAGACCGAACGC	used for validating co-transcription of *cmdABCDEF* genes
P78	GTAGAGCTGGAAGGGGTCGT<br>GAGAAGACCAACGACGACGC	used for validating co-transcription of *cmdABCDEF* genes
P89	CGGTAGAACGTGCGGTGGTT<br>ATCAGAACCCACAGCAGCCG	used for validating co-transcription of *cmdABCDEF* genes
P90	CCTTCGCCTCGTTCGCAGTA<br>AGAACGGCAAGGCCCCTAAG	used for validating co-transcription of *cmdABCDEF* genes
P01	CGAGGTCTTCGGCTTGTTGC<br>GACCGGCGTGTTCATCATCG	used for validating co-transcription of *cmdABCDEF* genes
PcmdB	GTTGCCGTTGACGTCCCAGA<br>TATGCCCAGAAGGCCGACGA	used to detect transcription level of *cmdB* gene
Pact	CTCATCGAGGAGTTGTGGGC<br>AGTTTGGCGTGCAGGGTCTC	used to detect transcription level of *actII-orf4*
P16S	TGCCAGCAGCCGCGGTAATA<br>GACTGCAGACCCGGGGTTAA	used to detect transcription level of *16S rRNA* gene
